# Distal Unfolding of Ferricytochrome c Induced by the F82K Mutation

**DOI:** 10.3390/ijms21062134

**Published:** 2020-03-20

**Authors:** Daniela Lalli, Camilla Rosa, Marco Allegrozzi, Paola Turano

**Affiliations:** 1 Magnetic Resonance Center (CERM) and Department of Chemistry, University of Florence, 50019 Sesto Fiorentino, Florence, Italy; daniela.lalli@uniupo.it (D.L.); camilla.rosa@meyer.it (C.R.); allegrozzi@cerm.unifi.it (M.A.); 2 Present Address: Dipartimento di Scienze e Innovazione Tecnologica, Università del Piemonte Orientale “A. Avogadro”, Viale T. Michel 11, 15121 Alessandria, Italy

**Keywords:** cytochrome c, redox-dependent ligand switch, distal site variants, alkaline transition

## Abstract

It is well known that axial coordination of heme iron in mitochondrial cytochrome c has redox-dependent stability. The Met80 heme iron axial ligand in the ferric form of the protein is relatively labile and can be easily replaced by alternative amino acid side chains under non-native conditions induced by alkaline pH, high temperature, or denaturing agents. Here, we showed a redox-dependent destabilization induced in human cytochrome c by substituting Phe82—conserved amino acid and a key actor in cytochrome c intermolecular interactions—with a Lys residue. Introducing a positive charge at position 82 did not significantly affect the structure of ferrous cytochrome c but caused localized unfolding of the distal site in the ferric state. As revealed by ^1^H NMR fingerprint, the ferric form of the F82K variant had axial coordination resembling the renowned alkaline species, where the detachment of the native Met80 ligand favored the formation of multiple conformations involving distal Lys residues binding to iron, but with more limited overall structural destabilization.

## 1. Introduction

**Figure 1:**
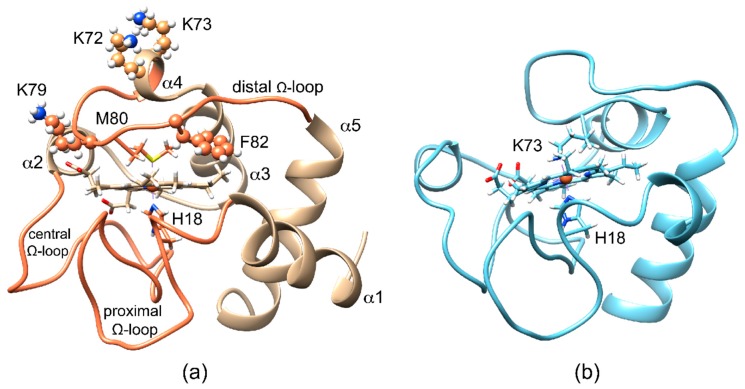
Cartoon representation of the backbone of mitochondrial cytochrome c (cyt c) with heme prosthetic group and iron axial ligands displayed as sticks. (**a**) The human cyt c (PDB id: 1J3S) under native conditions possesses a hexacoordinate heme iron, where His18 and Met80 as the axial heme iron ligands; the polypeptide fold consists of five α-helices and 3 Ω-loops (proximal, central, and distal); (**b**) the average structure of the NMR-determined family of the highly fluxional alkaline form of the K72AK79AC102T variant of yeast cyt c (PDB id: 1LMS), taken as a model of Lys73 axial-ligation.

Mitochondrial cytochrome c (cyt c) is a small globular heme protein (Figure 1a) with a His-Met axial ligation that confers high reduction potential to heme-iron, thus stabilizing the ferrous form [1]. In the ferric form, the Met80-iron coordination bond is relatively labile, giving rise to axial ligand switch under even slightly non-native conditions (relatively high temperature, low concentrations of denaturing agents or detergents, alkaline pH) [2,3,4,5,6,7,8]. As exemplified by the model structure of the alkaline form of yeast cyt c (Figure 1b), the release of Met80 bond is accompanied by a conformational reorganization of the 70-80 Ω-loop (distal Ω-loop), with the replacement of the distal Met ligand with Lys on the same loop [9]. The several Lys residues in close proximity of the heme (namely Lys 72, 73, and 79) on the distal side can indeed provide alternative donor atoms (Figure 1a). The oxidation state-modulated stability of the Met80-bond in the ferric protein is accompanied by a redox-dependent dynamics of the same area [10,11,12,13,14]. Recent reports have shown how axial ligand switch can also be induced under native conditions by destabilizing the H-bond network mediated by propionate-6, which acts as a stabilizing hinge to connect the distal Ω-loop to another Ω-loop, containing Thr48 and Thr49 (central Ω-loop) [15]. Noteworthy, the simple substitution of Met80 with an Ala residue at neutral pH gives rise to hydroxy-bound heme iron, thus suggesting the need for a further triggering effect to induce Ω-loop reorganization and ligand switch beyond the simple vacancy in heme iron coordination [16].

Among the residues on the distal Ω-loop, Phe82 is highly conserved [17]. Some of us have previously observed by NMR that substitution of the Phe82 residue with a Lys gives rise to a protein variant, F82K, with peculiar redox-dependent features [18]. X-ray crystallography, instead, has shown that replacing Phe82 with other hydrophobic residues in yeast ferrous cyt c does not alter the overall structure [19].

Here, we had deeper analyzed the structural and magnetic properties of the F82K variant by NMR, demonstrating that a Lys at position 82 could trigger distal ligand switch in human ferric cyt c. Ligand replacement was associated with structural variations localized on the distal loop and on helix α4, leaving the remaining structure substantially unaffected. To identify the new ferric distal ligand(s), a set of mutants carrying alanine residues in place of the potential iron-binding lysine residues was designed and characterized, namely, K79AF82K, K72AK73AF82K, and K72AK73AK79AF82K. The analysis of the hyperfine-shifted signals of the ^1^H NMR spectra of each mutant demonstrated the existence of complex equilibria among multiple protein conformations under slow exchange regimes on the NMR chemical shift time scale.

## 2. Results and Discussion

NMR was here employed to derive information on the F82K fold, on the presence of multiple conformational states, on the heme electronic structure, and on the effective magnetic moment of the human cyt c variants. In particular, structural modifications of cyt c induced upon substitution of Phe 82 with a Lys residue were monitored using ^1^H-^15^N Heteronuclear Single Quantum Coherence (HSQC) spectra that are sensitive to variations of the protein backbone conformation. In addition, the coordination sphere of the ferric iron in all variants—its electronic and spin states—and the presence of different conformers were inspected using paramagnetic one-dimensional (1D) ^1^H spectra that provided access to the hyperfine-shifted signals of the heme substituents and iron axial ligands, well-resolved outside the protein envelope. Finally, the effective magnetic moment of ferric F82K was determined by the modified Evans method [20].

### 2.1. Ferrous F82K Cyt C

The substitution of Phe82 with the bulky and charged Lys residue in reduced human cyt c yielded NMR spectra that clearly demonstrated the overall structural integrity and the retention of the heme iron axial coordination (Figure S1) [18]. The backbone resonance assignment of the reduced form of F82K cyt c, achieved through conventional multidimensional NMR triple resonance experiments, provided a detailed comparison with the wild type cyt c. Variations of amide-nitrogen and -proton resonances in ^1^H-^15^N HSQC maps were very small (<0.19 ppm) and confined to residues located on the distal side of the protein, in close proximity to the mutated amino acid (Figure S1). Even smaller perturbations (<0.12 ppm) were measured for Cys14, Gln16, and Thr19 that formed a cluster on the proximal side of the heme, mirroring Phe82 (Figure S1), as well as for Gly41, Arg91, Ala92, and Ile95. The well-resolved signals of Met80 side chain, a characteristic for ferrous cyt c, still resonated in the upfield part of the ^1^H spectrum of the reduced F82K mutant, although at slightly different chemical shift values (perturbation in the range −0.17 to +0.45 ppm) with respect to the wild type protein (Figure S2). These changes might be interpreted as due to the substitution with a Lys of a Phe residue, whose aromatic side-chain caused ring current shifts on nearby residues.

### 2.2. Ferric F82K Cyt C

**Figure 2:**
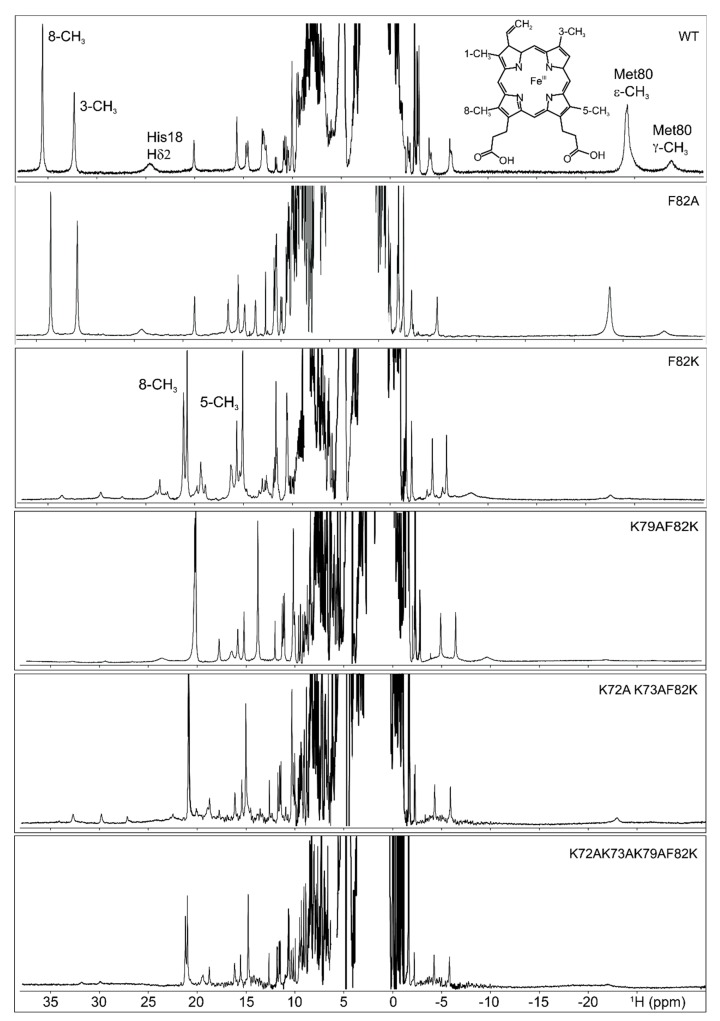
Comparison of the hyperfine shifted resonances in the ^1^H NMR spectra of the ferric form of the wild-type human cyt c and of its variants: F82A, F82K, K79AF82K, K72AK73AF82K, and K72AK73AK79AF82K. Spectra were acquired at 700 MHz and 298 K in 20 mM phosphate buffer, pH 6.8. The resolved heme methyl, His18 and Met80 signals for the wild type protein, and the 8-CH_3_ and 5-CH_3_ methyls for the F82K were provided. The structure of the heme prosthetic group of cyt c, where the methyl groups were numbered according to the Fischer nomenclature, is added as an inset.

The ^1^H NMR spectrum of ferric F82K showed two sets of hyperfine-shifted signals (Figure 2). One set was attributable to a minor form (about 4% of the population) with the typical NMR signature of wild type ferric cyt c (8-CH_3_ at 35.5 ppm, 3-CH_3_ at 32.3 ppm, measured at 298 K, downfield and upfield hyperfine shifted signals for His18 and largely upfield shifted Met80; Figure 2); the other set, which was ascribable to a major form, had a completely different pattern of hyperfine-resonances, with methyl signals resolved outside the diamagnetic envelope at approximately 21 ppm and 15.5 ppm at 298 K (Figure 2). Interestingly, the Met80 upfield-shifted signals resonating at −24.3 ppm and −28.8 ppm in the ^1^H spectrum of the wild type cyt c were non-detectable for the F82K mutant, suggesting the Met80 was displaced from iron coordination and substituted by other ligands. The detachment of the native distal ligand in ferric F82K was supported also by the CD spectrum of the oxidized mutant, where the weak negative CD band centered at about 415 nm [21], and diagnostic for the presence of the Met ligand was not visible. The downfield shifted signals of the major form of ferric F82K resembled in shift and line width those of non-native states of cyt c, where the Met80 was substituted by ligands with cylindrical symmetry [3,9,22] and, in particular, those of the alkaline state, where the native axial ligand was replaced by one of the distal Lys residues (Figure S3) [3,5,6,9,22,23,24]. More in detail, the set of broad hyperfine shifted signals at 23.4 ppm, 19.2 ppm, and 15.9 ppm looked similar to those of the coordinating Lys73 in alkaline yeast K79A cyt c (Figure S3) [9]. A close inspection of the spectra showed that the heme methyl signals of this major species were composed of multiple resonances with similar chemical shifts (Figure 2). The doubling of these resonances reminded the alkaline state of yeast wild type, where either Lys73 or Lys79 replace Met80 as axial ligand [22], and suggested the presence of multiple iron-binding lysine residues in the F82K variant, which generates different conformational states with similar magnetic properties and hyperfine shifts. Finally, the very broad features observed beneath the heme methyl resonances were similar in shift and line width to those assigned to the coordinating Lys73 in alkaline yeast K79A cyt c [9].

Replacement of Met80 by one or more distal Lys residues required a rearrangement of the protein structure, which particularly involved the distal Ω-loop (bearing Met80) and the preceding helix α4 (see Figure 1 to visualize these structural elements). Consistently, electron self-exchange between reduced and oxidized F82K via EXchange SpectroscopY (EXSY) 2D NMR revealed that the major form of the oxidized F82K was in slow exchange with both the reduced and the oxidized minor forms, which was consistent with the existence of a high energetic barrier for the conversion of the distal ligand. In contrast, the minor native-like form of oxidized F82K was in fast exchange with the reduced form, as commonly observed for the wild type protein [25], since no major structural changes occurred.

**Figure 3:**
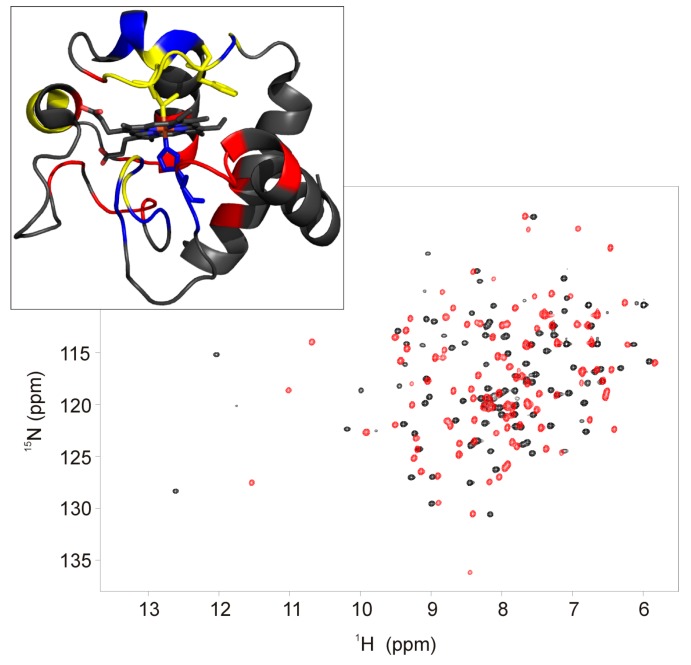
Overlay of the ^1^H-^15^N HSQC spectra of the ferric form of F82K (black trace) and wild type (red trace) human cyt c. Spectra were acquired at 800 MHz and 280 K in 20 mM phosphate buffer, pH 6.8. Residues of F82K showing chemical shift perturbation of the amide protons > 0.4 ppm and < −0.4 ppm with respect to the wild type cyt c are highlighted on the protein surface in blue and red, respectively (PDB id: 1J3S) (upper panel). Residues whose amide protons are not assigned are highlighted in yellow.

To interpret the observed hyperfine chemical shift pattern, the magnetic properties of ferric F82K were analyzed. The effective magnetic moment of ferric F82K, measured by the modified Evans method [20], was the same as for wild type cyt c (2.1 μB) and was invariant over the 280–300 K temperature range, as expected for a pure S = 1/2 spin state heme iron. The observed reduction in the extent of heme methyl ^1^H hyperfine shifts in ferric F82K should, therefore, originate from differences in magnetic susceptibility anisotropies (and, therefore, pseudo contact shift contributions) and/or differences in unpaired spin delocalization patterns, i.e., contact shifts [26]. Magnetic susceptibility anisotropies for His/Lys ligated cyt c have a slightly larger axial component with respect to the wild type protein (2.7 × 10^32^ m^3^ vs. 2.4 × 10^32^ m^3^) but a significantly smaller (in absolute value) rhombic component (−0.5 × 10^32^ m^3^ vs. 1.2 × 10^32^ m^3^) [13]. The same situation reasonably applied to ferric F82K. As most of the residues in wild type ferric cyt c experience non-negligible pseudo contact shifts [13,27,28], changes in the magnetic susceptibility anisotropy were expected to affect the chemical shift of most of the backbone amide resonances. As a matter of fact, the HSQC spectrum of ferric F82K resulted significantly different from that of the wild type protein (Figure 3), and its assignment was not straightforward because of the complications by the presence of multiple forms in slow exchange on the chemical shift time scale.

Using triple resonance experiments [29,30,31], 92.3% of the Cα, 90.4% of the CO, 91% of the backbone amides ^1^H and ^15^N, and 84.6% of the Cβ were sequence-specific assigned; the resonances of residues Lys53, Asn54, and Lys55 on helix α2, and of residues Lys79, Met80, Ile81, and Lys82 on the distal loop could not be identified (Figure 3). From a detailed NOEs (Nuclear Overhauser Effect) analysis, the sequential and medium-range connectivities (i.e., d_NN_ (i,i+1), d_NN_ (i,i+2), d_NN_ (i,i+3) and d_αN_ (i,i+1), d_αN_ (i,i+2), d_αN_ (i,i+3), and d_αN_ (i,i+4)) appeared conserved, demonstrating the integrity of the helices α1, α3, and α5. Even if the C-terminal part of helix α2 (residues 53-55) was not identified, a network of NOEs clearly showed that this secondary structure element was at least partially maintained; Thr49, located at the beginning of helix α2, showed a set of NOEs couplings with the sequential Ala51 and the following Ala52 and Asn53 residues (i.e., d_NN_ (i,i+1), d_NN_ (i,i+2), d_NN_ (i,i+3); d_βN_ (i,i+1), d_βN_ (i,i+2); d_γN_ (i,i+1), d_γN_ (i,i+2), d_γN_ (i,i+3)) consistent with the presence of one helical element. A set of NOEs detected between the residues Ile57 and Trp 59 situated in the C-term portion of the central Ω-loop connecting helix α2 to helix α3 and Leu35 and Thr40 (i.e., d_NN_ (40,59); d_Nδ1_ (40,57), d_Nδ1_ (59,35)) indicated that the structure of this loop (spanning residues 40-57) was substantially maintained with respect to the wild type cyt c. Residues 69-75, which formed helix α4 in the wild type cyt c, did not present the pattern of NOEs typical of the α-helices, thus suggesting that the structural rearrangement of the distal loop occurring upon detachment of the Met80 also involved the spatially close helix α4.

The backbone amides of Lys53, Lys55, Lys79, and Lys82 were not observed in the HSQC spectra, probably due to broadening beyond detection either due to paramagnetic effects (although in S = 1/2 heme systems such a situation can reasonably be encountered only for axial ligands) or, more probably, due to conformational equilibria between multiple protein conformations and chemical exchange with a bulk solvent, which might occur at intermediate rates on the NMR chemical shift time scale. The lack of signals for Lys 53 and Lys55 could be attributed to an increased solvent exposure associated with the distal structural rearrangement. 

### 2.3. F82A Cyt C

F82A was designed to verify the effect of Phe82 substitution with a neutral and hydrophobic residue on protein structure stability. In ferric F82A, the proton chemical shift of the heme methyl resonances, well-resolved outside the diamagnetic envelope, was the same as for the wild type protein (Figure 2). Proton chemical shift values were 33.47 ppm for 8-CH_3_ and 30.77 ppm 3-CH_3_ measured at 300 K, which compared well with 35.34 ppm and 32.05 ppm for the corresponding signals in the wild type protein measured at the same temperature. This observation clearly indicated that the substitution of Phe82 with a neutral and short-chain hydrophobic residue maintained the iron coordination sphere unaltered (at the level of both the proximal and the distal site) with respect to the wild type protein.

This behavior supported the idea that the structural destabilization characteristic of ferric F82K has an electrostatic origin. The introduction of a positively charged Lys residue in the distal side of the protein, in close proximity to the heme iron center (which results to be formally positively charged in the ferric form), generates an electrostatic repulsion that favores the replacement of the Met80 ligand by multiple Lys ligands. 

### 2.4. Additional Cyt C Variants

To investigate the origin of the observed multiple species observed in F82K spectra, a number of distal site axial mutants were prepared and characterized by NMR (Figure 2).

In order to identify which of the distal lysyl residues provide the sixth iron(III) ligand, all the amino acids known to substitute Met80 in iron coordination of the alkaline conformers of horse and yeast cyt c [22,23,32,33,34] were replaced by alanine residues. The candidate amino acids for mutation were Lys73, Lys72, and Lys79, leading to three new variants of cyt c: K79AF82K, K72AK73AF82K, and K72AK73AK79AF82K. Other Lys residues (53 and 55 belonging to helix α2 and 86, 87 and 88 belonging to helix α5) were not taken into account as candidate iron binders; their spatial distance from the metal ion would require very large conformational changes, which was not consistent with what observed in the NOE patterns, indicating an essentially unaltered central Ω-loop and α2 helix. Lys79 was first mutated into alanine to provide the K79AF82K double mutant, resembling one of the two conformers of the alkaline state [22]; then, Lys72 and Lys73 were substituted with alanine residues in single mutagenesis round to provide the K72AK73AF82K cyt c variant that resembled the second alkaline conformer [22]. Finally, all the three potential iron-binding Lys were replaced with alanine residues to provide the K72AK73AK79AF82K cyt c mutant.

The ^1^H NMR spectra of these mutants, reported in Figure 2, showed strong similarities among them. In the upfield region of the spectra, the same set of hyperfine-shifted resonances for His18 and Met80 was observed. In the downfield region of the spectra, the heme methyl resonances of the minor form resembled those of the ferric F82K. For the K79AF82K mutant, the minor species represented about 1% of the population, while, for the K72AK73AF82K and K72AK73AK79AF82K mutants, the 8% and the 4%, respectively. The signals of the heme methyl groups of the major form indicated the presence of three conformers in the K79AF82K cyt c mutant, where Lys72, Lys73, and/or Lys82 could provide the axial ligand. Only a single conformer was observed for the K72AK73AF82K, where either Lys79 or Lys82 could coordinate the heme iron. Two distinct sets of resonances were detected for the K72AK73AK79AF82K, where Lys82 represented the only possible heme iron ligand, suggesting that a conformational equilibrium between two Lys82-bound conformers occurred upon mutation of all the potential iron-binding Lys into Ala residues. The introduction of the Lys79 in the K72AK73AF82K mutant stabilized one of the two conformers. According to these observations, the heme methyl resonances of the major form of the ferric F82K could be interpreted as due to a mixture of different Lys-bound species, each of them possibly having more than one interconverting conformer, in equilibrium with the native form. Lys82, Lys79, Lys 73, and Lys72 might be all involved in heme iron-binding, providing axial ligands, and might modulate the affinity of the other Lys residues towards heme iron.

## 3. Materials and Methods

### 3.1. Site-Directed Mutagenesis

The plasmid pET-21a-CCHL-hCYC, which expresses human cyt c, was provided by Chuang and co-workers [35], and the mutated one, which expresses F82K, was already prepared in our laboratory [18]. New F82A, K79AF82K, K72AK73AF82K, K72AK73AK79AF82K mutants of human cyt c were obtained by mutating the pET-21a-CCHL-hCYC plasmid, as reported in the literature [18], using the Quick Change Site-Directed Mutagenesis Kit by Agilent Technologies and following the protocol provided therein. The F82A mutated plasmid was obtained with a 3-point mutation. The K79AF82K mutated plasmid was obtained with a single amino acid change (2 point mutations) on the F82K mutated plasmid; the K72AK73AF82K mutated plasmid was obtained with a double amino acid change (5 point mutations) on the F82K mutated plasmid; the K72AK73AK79AF82K mutated plasmid was obtained with a single amino acid change (2 point mutations) on the K72AK73AF82K mutated plasmid. All mutants were prepared with one mutant strand synthesis reaction each, using primers carrying all point mutations together. Gene sequencing to confirm mutations was performed (Primm Srl, Milano, Italy), and positive clones were used for protein expression.

### 3.2. Protein Expression and Purification

Human cyt c proteins were expressed, adapting protocols available in the literature [18,35]. Unlabeled F82K, F82A, K79AF82K, K72AK73AF82K mutants of human cyt *c* were expressed in minimal M9 medium containing 3.8 g L^−1^ glycerol as carbon source, supplemented with 1 mM MESNA (2-mercaptoethanesulfonic acid) and 0.1 mM δ-ALA (δ-aminolevulinic acid), two precursors of biosynthetic pathway to heme. A 100 mg L^−1^ FeSO_4_ solution was added to IPTG (IsoPropyl-β-D-ThioGalactopyranosid). Cells were grown for 72 h at 30 °C under slow shaking (60 rpm) after induction.

With respect to the above-described conditions, ^15^N,^13^C F82K with unlabeled heme was expressed using ^13^C glucose (2 g L^−1^) as carbon source instead of glycerol, and FeSO_4_ was not added to the medium. The use of the unlabeled precursors, coupled with the absence of FeSO_4_, guides the biosynthetic route towards heme unlabeling [36]. For K72AK73AK79AF82K, the method described for F82K, F82A, K79AF82K, and K72AK73AF82K mutants was not suitable because the protein could not be overexpressed. In order to produce an amount of protein sufficient for the preparation of an NMR sample, different conditions were tested, including changes in induction period, optimal temperature, and rate of shaking during expression. The unique positive result was obtained reaching a cell density with OD_600_ = 1.5 before induction by maintaining the same conditions, as for the method of the other two new mutants. However, the yield remained very poor (of the order of 2–3 mg L^−1^) but sufficient for recording 1D ^1^H NMR spectra. 

All the expressed protein variants were purified, as already reported in the literature [18].

### 3.3. NMR Sample Preparation

Typical protein concentration for NMR experiments was in the 0.2–1 mM range in 20 mM sodium phosphate buffer at pH 6.8, 10% D_2_O for the lock. The reduced state of the protein was obtained by the addition of DTT, while the oxidized one by the addition of an excess of K_3_Fe(CN)_6_, removed by ultrafiltration before measurements. 

### 3.4. Magnetic Susceptibility Measurements

According to standard procedures [20], coaxial NMR tubes were used with *ter*-butyl alcohol and 1,4-dioxane as internal references. The paramagnetic sample was prepared with 490 μM protein in 20 mM sodium phosphate buffer at pH 6.8, 10% D_2_O, and oxidized by the addition of an excess of ferricyanide, removed upon centrifugation; it was then transferred in the outer tube. The diamagnetic sample consisted of the ferrous cyt c and was transferred into the capillary inner tube. Then, the inner tube containing the diamagnetic solution was inserted into the outer tube containing the paramagnetic protein solution, and both were carefully capped. The shifts of the proton signals of the two reference molecules were measured at 280 K, 290 K, and 300 K at 700 MHz. The inner-outer tube peak separation (Δδ, measured in ppm) for each standard was assigned to the bulk susceptibility shift. The paramagnetic contribution to the bulk paramagnetic susceptibility was calculated according to the equations described in the literature [37].

### 3.5. High-Resolution NMR Experiments

One-dimensional ^1^H NMR spectra were used to observe the resonances of heme and axial ligands in ferrous and ferric forms of cyt c, thus probing the heme coordination sphere; ^1^H-^15^N HSQC spectra to derive information about backbone conformation; ^1^H-^1^H EXSY maps to explore possible exchange phenomena between different protein forms; triple resonance experiments (Table S1) to perform the sequential assignment of backbone nuclei (including Cβs). All the triple resonance NMR experiments of ^15^N,^13^C F82K sample were acquired at 298 K. The acquisition parameters for all these spectra are summarized in Table S1. NMR spectra were processed with Topspin version 2.0 and analyzed with the program Cara.

### 3.6. Assignment of F82K Cyt C

The backbone resonance assignment of the F82K cyt c variant was achieved through conventional multidimensional NMR triple resonance experiments, as summarized in Table S1.

## 4. Conclusions

It is well known that the ferric form of cyt c is more prone to the Met80 ligand switch than the ferrous form [6,24,38]. This behavior has been interpreted in terms of the reduced affinity of the harder iron(III) for the soft methionine sulfur donor atom [1]. Lower stability of the oxidized protein is paralleled by the larger mobility of its distal side [10,11,12,14]. The need for a triggering effect to induce the detachment of the distal ligand is interpreted as due to a release of the interactions that link α2 helix to the distal Ω-loop as well as the propionate-6-mediated interactions between the distal and central Ω-loops [15,27,39]. The model structure derived for the alkaline-unfolded form (Figure 1b) indicates an opening of the distal loop that promotes the coordination of distal lysines to iron [9]. On the other hand, the role of the central Ω-loop has received growing attention, given the discovery of modulation of apoptotic properties related to mutations and post-translational modification on this structural element [40,41,42,43,44,45].

The present results added further contributions to the above picture. Several cytochromes share with cyt c a Phe residue in a position homologous to that of Phe82 in mitochondrial cyt c [46,47]. Still, its conservation results to be more a functional requirement rather than stability need: Ser, Trp, or Ala residues are equally efficient in terms of structure stabilization at neutral pH [48,49]. Introducing a Lys has no effects on the ferrous protein, where the heme iron center results formally neutral, but it causes dramatic effects on the structure of the ferric form, which has a metal center with a formal charge of +1 [50]. The behavior can be interpreted in terms of a very delicate electrostatic balance: the positively charged iron center in oxidized cyt c cannot bear a further positive charge at the distal side, where several Lys residues are already present. An electrostatic repulsion occurs between the metal ion and the Lys side chain hanging from position 82, and most probably pointing towards the heme center, which facilitates the opening of the distal Ω-loop, as observed for the wild type protein at alkaline pH, but instead leaving essentially unaltered the central Ω-loop and helix α2.

Learning about the stability of the Met80-Fe(III) bond is relevant for a better knowledge of the structural events that lead to cyt c release into the cytosol for initiating the apoptotic cascade. The formation of a heterogeneous ensemble of non-native protein species, where the Met80-Fe(III) bond is lost, characterizes the interaction between cyt c and cardiolipin, with the involvement of Lys72, 73, and 79 [51], and destabilization of the Tyr67-Met80-Phe82 hydrophobic cluster. The present work, although not specifically referring to the cardiolipin-cyt c interaction, shed further light on the delicate interplay among the residues in the distal site that allows cyt c to “decide” between a pro-survival electron transfer activity or an anti-survival apoptotic function.

## Supplementary Materials

Supplementary materials can be found at https://www.mdpi.com/1422-0067/21/6/2134/s1.

## Abbreviations

NMR	Nuclear Magnetic Resonance
Cyt c	Cytochrome c
EXSY	EXchange SpectroscopY
HSQC	Heteronuclear Single Quantum Coherence
CD	Circular Dichroism
MESNA	2-Mercaptoethanesulfonic Acid
IPTG	IsoPropyl-β-D-ThioGalactopyranosid
OD	Optical Density
