# Supplementary material for: Distal Unfolding of Ferricytochrome c Induced by the F82K Mutation
Source: Int J Mol Sci. 2020 Mar 20;21(6):2134. doi: 10.3390/ijms21062134 (PMC7139943; doi:10.3390/ijms21062134)
Supplement: Supplementary file 1 [file ijms-21-02134-s001.pdf]

International Journal of
Molecular Sciences

Supplementary Material

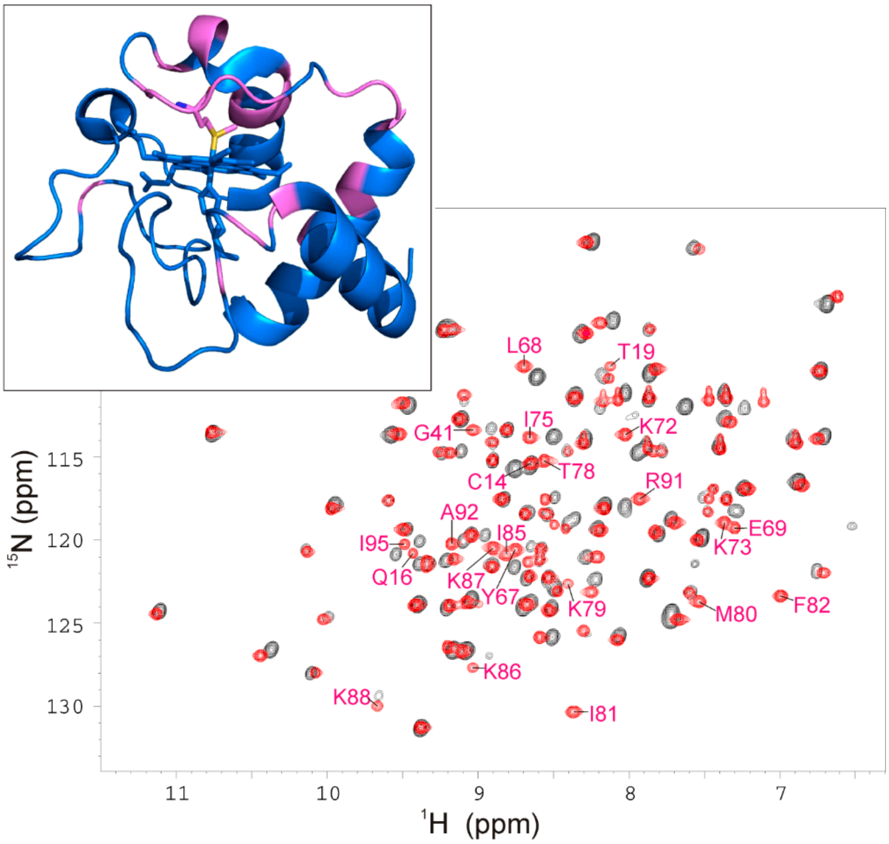

Figure S1. Superimposition of the ^{1}H-^{15}N HSQC spectra of the ferrous forms of F82K (black trace) and wild type (red trace) human cyt c. Spectra were acquired at 500 MHz and 298 K, in 20 mM phosphate buffer, pH 6.8. Residues of F82K showing an average chemical shift perturbation > 0.06 ppm with respect to the wild type cyt c are labelled in the spectra and highlighted in magenta on the protein surface (PDB id: 1J3S) (upper panel).

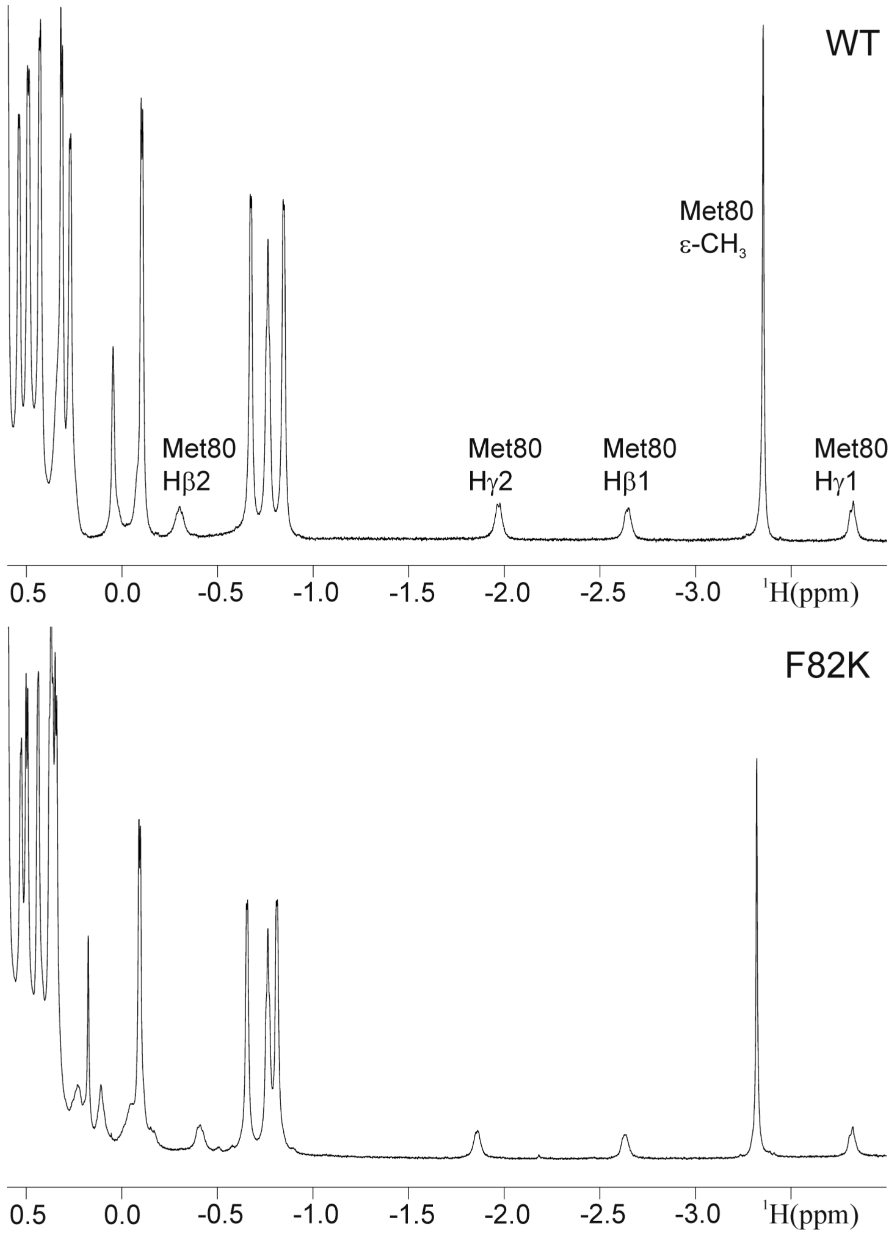

Figure S2. Comparison of the upfield region of the ^{1}H NMR spectra acquired on the ferrous form of wild type and F82K human cyt c. The Met80 side chain resonances are labeled for the wild type protein according to the available assignment. Spectra were acquired at 800 MHz and 300 K, in 20 mM phosphate buffer, pH 6.8.

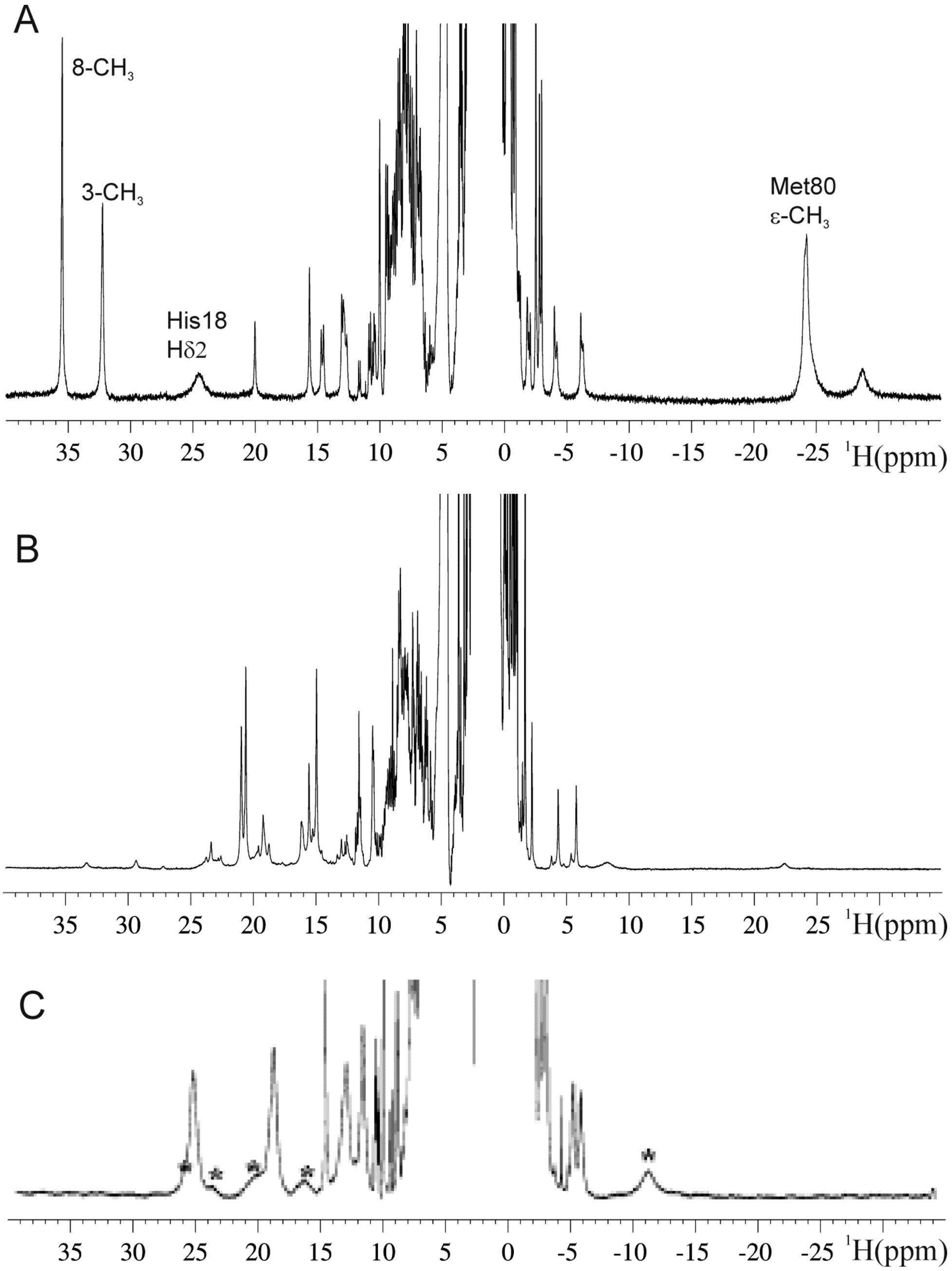

Figure S3. Comparison of the hyperfine shifted resonances in the ^{1}H NMR spectra of the ferric form of (A) wild type, and (B) F82K human cyt c. Spectra were acquired at 700 MHz and 298 K, in 20 mM phosphate buffer, pH 6.8. The resolved heme methyl, His18 and Met80 signals are labeled for the wild type protein according to the available assignment. In panel C, the spectrum of the alkaline form of K79A yeast cytochrome c (taken from ref.[1]) is reported for comparison purposes; signals of the Lys ligand(s) are marked with asterisks.

Table S1. Main NMR experiments.

**Experiments**	**Dimension of acquired data (nucleus)**			Spectral width (ppm)		
	**t_1**	**t_2**	**t_3**	F_1	F_2	F_3
^{1}H 1D^{a,b}	32768	-	-	100	-	-
^{1}H-^{15}N HSQC^{b}	256 (^{15}N)	1024 (^{1}H)	-	40.0	18.0	-
^{1}H-^{1}H EXSY^{b}	512 (^{1}H)	1024 (^{1}H)	-	70.0	70.0	-
HNCA^{b}	128 (^{13}C)	48 (^{15}N)	1024 (^{1}H)	40.0	40.0	18.0
HN(CO)CA^{b}	128 (^{13}C)	48 (^{15}N)	1024 (^{1}H)	40.0	40.0	18.0
HNCO^{b}	128 (^{13}C)	48 (^{15}N)	1024 (^{1}H)	20.0	40.0	18.0

CBCA(CO)NH[b]	128 (^{13}C)	48 (^{15}N)	1024 (^{1}H)	80.0	40.0	18.0
HNCACB[b]	128 (^{13}C)	48 (^{15}N)	1024 (^{1}H)	80.0	40.0	18.0

a Spectra acquired at 700 MHz equipped with cryo-probe (TXI 5-mm); b Spectra acquired at 800 MHz equipped with cryo-probe (TXI 5-mm).

References:

1. Assfalg, M.; Bertini, I.; Dolfi, A.; Turano, P.; Mauk, A. G.; Rosell, F. I.; Gray, H. B., Structural model for an alkaline form of ferricytochrome C. *J. Am. Chem. Soc.* **2003,** *125*, 2913–2922.

© 2019 by the authors. Submitted for possible open access publication under the terms and conditions of the Creative Commons Attribution (CC BY) license (http://creativecommons.org/licenses/by/4.0/).
